# Elevated Levels of Neutrophil Activated Proteins, Alpha-Defensins (DEFA1), Calprotectin (S100A8/A9) and Myeloperoxidase (MPO) Are Associated With Disease Severity in COVID-19 Patients

**DOI:** 10.3389/fcimb.2021.751232

**Published:** 2021-10-21

**Authors:** Shubham Shrivastava, Shweta Chelluboina, Prashant Jedge, Purwa Doke, Sonali Palkar, Akhilesh Chandra Mishra, Vidya A. Arankalle

**Affiliations:** ^1^ Department of Communicable Diseases, Interactive Research School for Health Affairs (IRSHA), Bharati Vidyapeeth (Deemed to be University), Pune, India; ^2^ Department of Critical Care Medicine, Bharati Vidyapeeth (Deemed to be University) Medical College, Pune, India; ^3^ Department of Medicine, Bharati Vidyapeeth (Deemed to be University) Medical College, Pune, India; ^4^ Department of Community Medicine, Bharati Vidyapeeth (Deemed to be University) Medical College, Pune, India

**Keywords:** COVID-19, neutrophil, alpha-defensin, disease severity, S100A8/A9, mechanical ventilation, secondary infection, biomarker

## Abstract

Understanding of the basis for severity and fatal outcome of SARS-CoV-2 infection is of paramount importance for developing therapeutic options and identification of prognostic markers. So far, accumulation of neutrophils and increased levels of pro-inflammatory cytokines are associated with disease severity in COVID-19 patients. In this study, we aimed to compare circulatory levels of neutrophil secretory proteins, alpha-defensins (DEFA1), calprotectin (S100A8/A9), and myeloperoxidase (MPO) in COVID-19 patients with different clinical presentations. We studied 19 healthy subjects, 63 COVID-19 patients with mild (n=32) and severe (n=31) disease, 23 asymptomatic individuals identified through contact tracing programme and 23 recovering patients (1-4 months post-disease). At the time of disease presentation, serum levels of DEFA1 were significantly higher in patients with mild (mean230 ± 17, p<0.0001) and severe (mean452 ± 46, p<0.0001) disease respectively in comparison to healthy subjects (mean113 ± 11). S100A8/A9 proteins were significantly higher in COVID-19 patients (p<0.0001) irrespective of disease severity. The levels of DEFA1, S100A8/A9 and MPO reduced to normal in recovering patients and comparable to healthy subjects. Surprisingly, DEFA1 levels were higher in severe than mild patients in first week of onset of disease (p=0.004). Odds-ratio analysis showed that DEFA1 could act as potential biomarker in predicting disease severity (OR=11.34). In addition, levels of DEFA1 and S100A8/A9 were significantly higher in patients with fatal outcome (p=0.004 and p=0.03) respectively. The rise in DEFA1 levels was independent of secondary infections. In conclusion, our data suggest that induction of elevated levels of alpha-defensins and S100A8/A9 is associated with poor disease outcome in COVID-19 patients.

## Introduction

COVID-19 is a recently emerged viral disease caused by a betacoronavirus, SARS-CoV-2. The viral infection has spread globally in a short span of time affecting 196 million people and causing approximately 4.2 million deaths as of 29^th^ July 2021 (John Hopkins University Coronavirus tracker).

Majority of the infected individuals exhibit either no or mild symptoms and about 15% develop severe pneumonia with approximately 6% progressing to acute respiratory distress syndrome (ARDS) and multiorgan failure (Guan et al., 2020). Inflammatory cytokines, IL-6, IL-10 and TNF-alpha have been identified as independent predictors of disease severity and clinical outcome (Del Valle et al., 2020; Dhar et al., 2021). Even, spike protein of SARS-CoV-2 is reported to induce inflammatory cytokines in macrophages (Khan et al., 2021). One of the hypotheses for progression to serious disease complications is imbalanced host immune response with ineffective early innate immunity followed by impaired adaptive immune responses and hyper-inflammation (Blanco-Melo et al., 2020; Tay et al., 2020).

Several studies reported alterations in both innate and adaptive arms of the host immunity against SARS-CoV-2 infection in COVID-19 patients (Diao et al., 2020; Zhang et al., 2020; Zheng et al., 2020; Munje et al., 2021). During airway infections, neutrophils are the first line of defense. In response to viral infection, neutrophils migrate to the site of infection and initiate the antiviral defense mechanism. Recent studies have reported accumulation of neutrophils in severe COVID-19 patients compared to non-severe patients (Huang et al., 2020; Kuri-Cervantes et al., 2020; Liao et al., 2020; Tan et al., 2020; Wang D. et al., 2020). In a COVID-19 patient with fatal outcome, large number of neutrophils were found in autopsied lung infiltrates (Barnes et al., 2020). Moreover, increased neutrophil to lymphocyte ratio in peripheral blood of severe COVID-19 patients (Zhang et al., 2020) suggest that neutrophil activation may modulate immune response after SARS-CoV-2 infection. Upon neutrophil activation or death, alarmins including alpha-defensins, S100A8/A9 heterodimers (calprotectin) and myeloperoxidase (MPO) proteins are released extracellularly in circulation (Faurschou and Borregaard, 2003; Tardif et al., 2015). Till date, COVID-19 pathogenesis has focused on macrophages, T and B cells. It has been suggested that neutrophilia is associated with disease severity in COVID-19. However, it is still unknown whether neutrophils drive the inflammation or act as bystanders. In this study, we aimed to quantitate the circulatory levels of neutrophil’s secretory proteins in different clinical presentations of COVID-19 patients.

## Methods

### Study Design and Participants

This study was approved by the Institutional Ethics committee of Bharati Vidyapeeth (Deemed to be University) Medical College and Hospital, Pune (IEC/2020/25). The study was conducted using 125 serum or plasma samples collected during May-September 2020 from 109 SARS-CoV-2 RNA positive COVID-19 patients. The study included: (1) Nineteen healthy volunteers negative for anti-SARS-CoV-2 IgG antibodies (2) Sixty-three hospitalized patients with confirmed COVID-19 disease sampled within 4 weeks of illness. These were further classified as mild (n=32) or severe (n=31) infections based on disease severity (WHO 2020) (3) 23 asymptomatic individuals identified among contacts of COVID-19 patients and (4) 23 COVID-19 patients during convalescence (1-4 months post disease onset). After informed consent, approximately 5 ml blood was collected at the time of admission from all the hospitalized patients. Follow-up blood samples were collected within 2 weeks after admission from 9 mild patients. Sequential blood samples were also collected within one week from 7 patients with severe disease. Serum or plasma samples were stored in aliquots at -80°C for further use.

### DEFA1 and S100A8/A9 ELISA

Levels of alpha defensins, DEFA1 and S100A8/A9 in serum and plasma were quantified using commercially available ELISA kits, Neutrophil DEFA1 (USCN, Wuhan) & quantikine ELISA for human S100A8/A9 Heterodimer (R & D systems, Minneapolis) as per manufacturer’s protocols. The minimum detection limits for DEFA1 & S100A8/A9 were 20ng/ml & 40ng/ml respectively.

### Myeloperoxidase Assay

Serum/plasma samples were diluted with 1X PBS in the ratio of 1:5. The diluted samples were then incubated with 0.75mM hydrogen peroxide solution (Sigma-Aldrich, St Louis, MO) and 1.6mM tetramethylbenzidine (TMB) solution (Sigma) for 10 mins. Myeloperoxidase levels were measured on the basis of OD values (450nm) obtained after the addition of TMB solution and subsequent stoppage of the reaction with sulphuric acid and absorbance was measured at 450nm.

### Statistical Analysis

Statistical analysis was performed using Graph Pad Prism 5.0 software. Mann‐Whitney U test was used to compare the data between the study groups. Paired samples were analyzed using Wilcoxon signed rank test. Receiver operating characteristic (ROC) curves were generated to determine the predicted value of alpha-defensin as biomarker. The area under the curve (AUC) and 95% confidence interval (CI) were calculated.

## Results

**Table 1** summarizes the demographic features and hematological/biochemical parameters among the COVID-19 patients enrolled in this study. Of the 109 patients studied, 23 were viral RNA positive asymptomatic contacts of COVID-19 index cases while 63 patients were symptomatic with mild (n=32) or severe (n=31) disease. Among 31 patients suffering from severe disease, 19 (61.3%) required mechanical ventilation for oxygen support and 11 (35.5%) succumbed to the infection.

**Table 1:** Details of the COVID-19 patients examined at presentation.

Characteristics*	Healthy (n = 19)	Asymptomatic (n = 23)	Mild (n = 32)	Severe (n = 31)	Convalescent (n = 23)	p-value^$^
Age in years (median)^#^	27 (18-33)	46 (6-70)	40 (15-67)	59 (30-85)	26 (15-51)	
Male (%)	10 (53%)	12 (52%)	19 (59%)	21 (68%)	16 (70%)	
Female (%)	9 (47%)	11 (48%)	13 (41%)	10 (32%)	7 (30%)	
Mechanical Ventilation	NA	NA	NA	19 (61.3%)	NA	
Leukocyte count (per mm^3^)	–	–	6205 ± 416	9183 ± 961	–	0.007
% of neutrophils	–	–	61 ± 3	80 ± 2	–	<0.0001
% of lymphocytes	–	–	29 ± 2	13 ± 2	–	<0.0001
Neutrophil to lymphocyte ratio	–	–	3 ± 1	12 ± 3	–	0.007
Platelets count	–	–	237909 ± 14504	241300 ± 21294	–	0.89
D-dimer (ng/mL)	–	–	198 ± 38	984 ± 258	–	0.005
CRP (mg/L)	–	–	34 ± 13	206 ± 39	–	0.0002
DEFA1 (ng/mL)	113 ± 11	177 ± 44	230 ± 17	452 ± 46	175 ± 36	<0.0001
S100A8/A9 (Calprotectin, ng/mL)	2288 ± 687	10134 ± 1236	16589 ± 2651	18183 ± 2512	4645 ± 899	0.48

*Values are expressed as mean ± SEM; ^#^age in years expressed as median (range). NA denotes as not applicable. –indicates not available. ^$^indicates statistical significance on comparison of datasets between mild and severe groups.

### Expression Levels of Alpha-Defensins (DEFA1), S100A8/A9 and MPO Proteins in COVID-19 Patients

**Figure 1:**
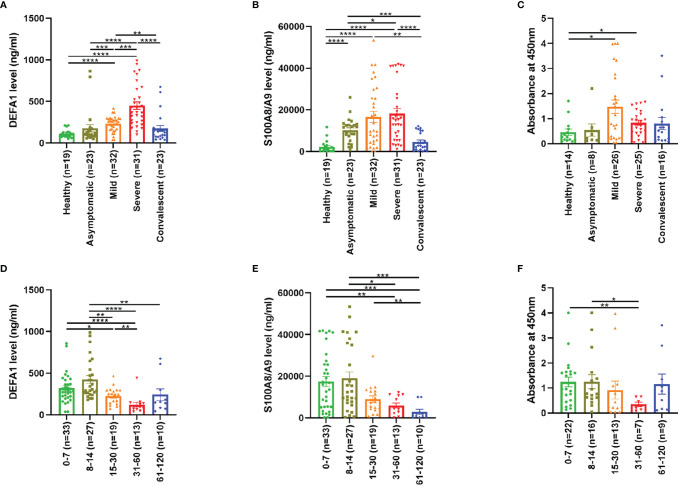
Expression levels of neutrophil activated proteins in sera/plasma of COVID-19 patients. Levels of **(A)** DEFA1, **(B)** S100A8/A9 and **(C)** MPO in COVID-19 patients with different clinical presentations in comparison to healthy controls. Figures D-F depict modulation of **(D)** DEFA1, **(E)** S100A8/A9 and **(F)** MPO levels in COVID-19 patients on different days post onset of disease (POD). The data is presented as dot plots with bar representing the mean ± SEM in each group. Each dot represents a single sample. P values were calculated using Mann-Whitney test. * denotes p-value < 0.05, ** denotes p-value < 0.01, *** denotes p-value < 0.001 and **** denotes p-value < 0.0001.

**Figure 1** depicts circulatory levels of (A) DEFA1, (B) S100A8/A9 and (C) MPO in serum/plasma samples of COVID-19 patients. As compared to the healthy subjects (mean 113±11), a significant increase in alpha-defensins (DEFA1) was recorded in the patients with mild (mean 230±17, p<0.0001) or severe disease (mean 452±46, p<0.0001, **Figure 1A**). Importantly, the rise was more pronounced in the patients with severe disease than those with mild infection (p=0.0001). Asymptomatic individuals were comparable to healthy controls (p=0.53). During convalescence, a significant drop was evident in DEFA1 levels from the patients with mild (p=0.004) and severe (p<0.0001) infections. At this time, alpha-defensins levels returned to normal levels comparable with the healthy controls (p=0.49).

Irrespective of disease severity, the levels of S100A8/A9 were elevated in all the individuals with asymptomatic (p<0.0001) and symptomatic (p<0.0001) infections, when compared to the healthy controls (**Figure 1B**). However, the rise was not significantly different among symptomatic infections with mild or severe disease (p=0.48). During convalescence, the levels of S100A8/A9 were comparable to healthy controls (p=0.11).

In a subset of samples, MPO levels were determined (**Figure 1C**). Similar to DEFA1, MPO levels were unaltered during subclinical infection (p=0.85) while a significant increase was observed in the patients with mild (p=0.01) and severe (p=0.02) disease. However, the difference with respect to disease severity was not significant (p=0.29). During convalescence, MPO levels were comparable to healthy controls (p=0.42).

To understand the dynamics of neutrophil secretory proteins in COVID-19 patients, expression levels of DEFA1, S100A8/A9 and MPO proteins were compared at different time points post onset of disease (POD) (**Figures 1D–F**). During the first week of the disease, levels of DEFA1, S100A8/A9 and MPO proteins were elevated that remained higher during the second week. Subsequently, significant decline was seen in all the three proteins. This suggests that neutrophil remained activated till 14 days post infection and returned back to resting state by 15-30 days post infection.

### Expression Levels of DEFA1, S100A8/A9 and MPO Proteins in Relation to Disease Severity in COVID-19 Patients

**Figure 2:**
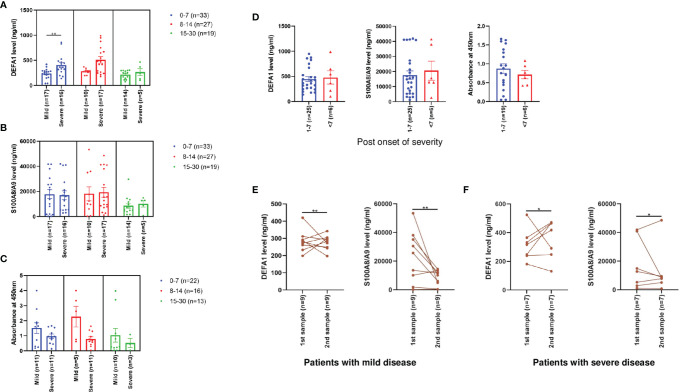
Expression levels of neutrophil activated proteins in sera/plasma of mild and severe COVID-19 patients. Figures A-C depict comparisons of levels of **(A)** DEFA1, **(B)** S100A8/A9 and **(C)** MPO among mild and severe disease patients at different PODs. Comparative levels of **(D)** DEFA1, S100A8/A9 and MPO among severe disease patients at different days post onset of severity. The data is presented as dot plots with bar representing the mean ± SEM in each group. Each dot represents a single sample. P values were calculated using Mann-Whitney test. ** denotes p-value < 0.01. **(E)** Expression levels of DEFA1 and S100A8/A9 levels in SARS-CoV-2 patients with mild disease at the time of admission (POD 2-14) and at follow-up (POD 8-21). **(F)** Expression levels of DEFA1 and S100A8/A9 levels in SARS-CoV-2 patients with severe disease at the time of admission (POD 5-14) and at follow-up (POD 7-19).The data are presented as line graphs with each line representing a single individual. P values were calculated using the Wilcoxon signed rank test. * denotes p-value < 0.05, ** denotes p-value < 0.01.

Next, the levels of neutrophil secretory proteins with respect to disease severity and PODs were compared (**Figures 2A–C**). Interestingly, DEFA1 expression levels were significantly higher in patients with severe disease during the first week itself (p=0.004). No significant difference was observed during the second (p=0.052) and the third (p=0.97) weeks post onset of disease. S100A8/A9 and MPO levels did not differ among mild and severe disease patients at different time points post onset of disease.

To understand whether the increased expression of neutrophil secretory proteins is associated with the onset of severity, the levels of these proteins were compared with respect to the day on which severity was first identified. Of the 31 severe disease patients, 16 (52%), 9 (29%) and 6 (19%) were respectively collected within 0-3, 4-7 and 8-23 days of the identification of severity. Expression levels of all the three proteins were elevated within 7 days post identification of severe disease (**Figure 2D**) suggesting that higher levels of neutrophil secretory proteins are associated with disease severity in COVID-19 patients.

Next, DEFA1 and S100A8/A9 levels were determined in patients with mild (n=9) and severe disease (n=7) at two time points. For patients with mild disease, first sampling was done at the time of admission (POD 2-14) and second sampling was done at 6-12 days later (POD 8-21). An increase in DEFA1 levels was evident in the mild disease (p=0.004, **Figure 2E**). On contrary, levels of S100A8/A9 decreased in 7 out of 9 mild patients (p=0.004, **Figure 2E**). For patients with severe disease, the duration between first (POD 5-14) and second (POD 7-19) sampling was 2-5 days. During the follow up, DEFA1 levels increased in 5/7 patients (p=0.02, **Figure 2F**) while a decrease in S100A8/A9 levels was recorded in 4/7 patients (p=0.02, **Figure 2F**).

### Expression Levels of DEFA1, S100A8/A9 and MPO Proteins in Relation to Disease Severity and Mechanical Ventilation/Secondary Infection

**Figure 3:**
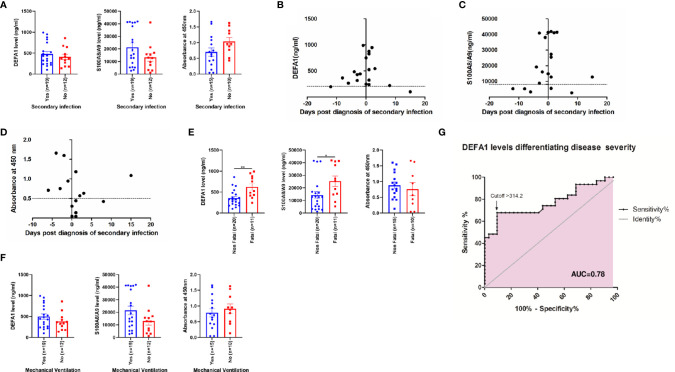
Expression levels of neutrophil activated proteins in severe COVID-19 patients in relation to occurrence of secondary infection and mechanical ventilation. Figure **(A)** depict comparative levels of DEFA1, S100A8/A9 and MPO in severe patients with or without secondary infection. The data is presented as dot plots with bar representing the mean ± SEM in each group. Each dot represents a single sample. P values were calculated using Mann-Whitney test. Figures **(B–D)** depicts levels of **(B)** DEFA1, **(C)** S100A8/A9 and **(D)** MPO on different days before or after diagnosis of secondary infection. Each dot represents a single sample. The dotted line indicates cut-off value calculated as average value of expression levels of neutrophil activated proteins in healthy controls + 2*standard deviation. Comparative levels of DEFA1, S100A8/A9 and MPO among severe disease patients with **(E)** non-fatal and fatal disease outcome and **(F)** requiring mechanical ventilation. The data is presented as dot plots with bar representing the mean ± SEM in each group. Each dot represents a single sample. P values were calculated using Mann-Whitney test. * denotes p-value < 0.05, ** denotes p-value < 0.01. **(G)** Receiver-operator characteristics (ROC) curve of DEFA1 serum levels for the prediction of COVID-19 disease severity among mild and severe disease presentations.

In view of the fact that a large proportion of severe disease patients require mechanical ventilation and develop secondary infections, further analyses were undertaken to assess the contribution of these confounding factors in the observed rise of neutrophil secretory proteins. In our series, ~95% of the severe patients undergoing intubation developed secondary infection. As evident from **Figure 3**, the expression levels of all the three proteins were similar in both the groups (**Figure 3A**). We further compared the expression of all the three proteins in relation to the duration between the day of secondary diagnosis and sampling (**Figures 3B–D**). Clearly, high expression of these proteins was not related to secondary bacterial infection but the outcome of SARS-CoV-2 infection.

Next, we compared the expression levels of neutrophil secretory proteins in the patients with severe disease in relation to the disease outcome (**Figure 3E**). DEFA1 and S100A8/A9 expression levels were significantly higher in patients with fatal outcome (p=0.004; p=0.03 respectively) suggesting that neutrophil activation is associated with mortality in COVID-19 patients. No difference in the levels of MPO protein was observed (p=0.56). Comparison of these proteins in severe patients requiring mechanical ventilation revealed that the rise was independent of the use of mechanical ventilator for oxygen support (**Figure 3F**). Furthermore, odds-ratio analysis revealed that poor outcome of the disease was strongly associated with the requirement of mechanical ventilation by the patients suffering from severe illness (OR = 12).

To examine the possible contribution of mechanical ventilation or secondary infection in raising the DEFA1 levels in severe patients, a separate analysis was undertaken. DEFA1 levels were significantly higher when patients with severe disease without these confounding factors (n=11; 385±65 ng/mL) were compared patients with mild disease (n=32; 230±17 ng/mL, p=0.02). Thus, higher expression of DEFA1 during severe COVID-19 infection was independent of mechanical ventilation or secondary infection.

### DEFA1 as Potential Biomarker for Severity

To explore, if DEFA1 levels can be used to differentiate between mild and severe disease presentations, three different concentrations of DEFA1 were evaluated (250, 300 and 350 ng/mL). At 250ng/mL, DEFA1 could differentiate between mild and severe disease presentations (OR = 3.68). At 300 ng/mL DEFA1 concentration, odds-ratio increased to 11.34 and at 350ng/mL DEFA1 concentration, odds ratio further increased to 13.38. ROC curve analysis revealed that at 314 ng/mL, the estimated sensitivity and specificity were 68% and 91% respectively (**Figure 3G**). The AUC value for alpha-defensin was 0.78 ± 0.06 (95% CI = 0.66 to 0.89, p<0.0002), suggesting that DEFA1 levels could act as a potential biomarker in predicting disease severity in COVID-19.

### Correlation Analysis Among Different Parameters

**Figure 4:**
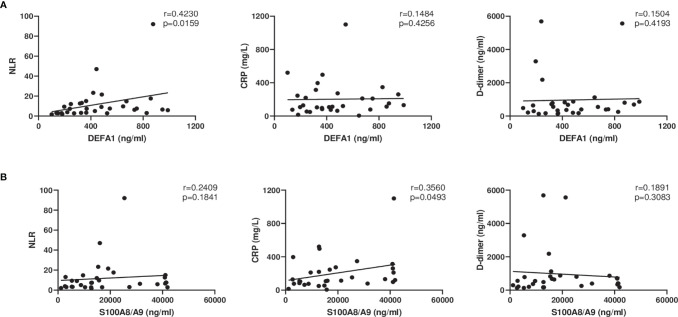
Correlation analyses between levels of DEFA1 and S100A8/A9 with clinical laboratory parameters in COVID-19 patients with severe disease at the time of admission. **(A)** DEFA1 levels compared with Neutrophil to Lymphocyte Ratio (NLR), C reactive protein (CRP), D-dimer levels. **(B)** Levels of S100A8/A9 compared with NLR, CRP and D-dimer levels. Spearman’s correlation coefficient was calculated for each graph.

Certain clinical parameters such as Neutrophils to Lymphocytes ratio (NLR), C-reactive protein (CRP) and D-dimer have been associated with severity of COVID-19 (Chen et al., 2020; Liu et al., 2020). We attempted to correlate these parameters with known significance with the proteins evaluated in this study (**Figure 4**). DEFA1 levels were found to be positively correlated with NLR (r=0.42, p=0.02). S100A8/A9 expression levels were found to be positively correlated with CRP (r=0.36, p=0.05).

## Discussion

This study provides comparative, circulatory expression profiles of DEFA1, S100A8/A9 and MPO in COVID-19 patients with different clinical presentations. Though SARS-CoV-2 infection involves neutrophil activation, a marked difference was noted in the association of these markers with disease severity. Of note, raised DEFA1 and MPO levels characterized symptomatic infections while elevated levels of calprotectin marked SARS-CoV-2 infections irrespective of clinical presentation (**Figure 1**). Further, MPO and calprotectin levels did not differ among patients with mild or severe disease. An important observation was a significant rise in DEFA1 levels during the first week post-disease onset in severe than non-severe patients (**Figure 2A**). Odds-ratio analysis showed that DEFA1 could act as potential biomarker in predicting disease severity (OR=11.34). Of note, at 314 ng/mL DEFA1 levels, accurate prediction of disease severity in differentiating mild from severe disease patients could be determined with 68% sensitivity and 91% specificity (**Figure 3G**). This significant observation with small numbers need to be extended to a larger cohort with prospective follow-up studies.

Single cell RNA sequencing analyses showed the increased presence of dysfunctional neutrophils expressing S100A8, S100A9, DEFA3 and DEFA4 genes in the PBMCs of severe COVID-19 patients suggesting association of the phenotypic alterations in neutrophils with disease severity (Schulte-Schrepping et al., 2020). Very recently, increased serum DEFA1 levels were reported in COVID-19 patients suffering from acute respiratory distress syndrome (Kerget et al., 2021). Other clinical presentations were not studied. Contrary to the biomarker role of alpha-defensins, antiviral role of DEFA1 was established in *in-vitro* wherein HNP1 inhibited the viral infection at the step of viral entry by disrupting the fusion of the SARS-CoV-2 virus with host membrane (Xu et al., 2021). Similarly, antiviral activity of intestinal alpha-defensin, HD-5 released from Paneth cells and of β-defensin, hBD-2 was shown to be due to blocking of the entry of spike expressing pseudovirus into ACE2 expressing cells (Wang C. et al., 2020; Zhang et al., 2021). At the site of infection, neutrophils can exert their antiviral activities by increasing the expression of peptides such as, defensins and promote degranulation. However, excessive release of defensins and neutrophil elastase led to cell death of bronchial epithelial cells thereby increasing permeability of blood vessels and causing acute lung injury (Grommes and Soehnlein, 2011). The mechanism(s) by which defensins contribute to disease severity during SARS-CoV-2 infection are not yet known.

Elevated calprotectin levels in the blood of COVID-19 patients with severe disease are consistent with a study from Michigan, USA (Shi et al., 2020). In addition, we report that elevation of calprotectin was a function of SARS-CoV-2 infection and was not related to disease severity. The observation of higher expression of S100A8 and S100A9 genes in the PBMCs of the patients with severe disease but not in patients with mild disease is noteworthy (Silvin et al., 2020). The discrepancy between gene expression and protein levels (this study) could be due to differences in sampling timings, increase only at transcription level or transient rise in the circulatory proteins missed by our study. In children suffering with multisystem inflammatory syndrome (MIS-C) after SARS-CoV-2 infection, rise in S100 protein levels was similar to other inflammatory disorder such, as Kawasaki disease (Rodriguez-Smith et al., 2021). Disease severity was associated with increased, circulatory S100B protein levels as well (Aceti et al., 2020).

So far, several multi-omics studies revealed that genes involved in neutrophil function and activation are highly upregulated and neutrophil degranulation is the most prominent defense pathway in SARS-CoV-2 infected patients (Akgun et al., 2020; Schulte-Schrepping et al., 2020; Bankar et al., 2021). Elevated levels of MPO in nasopharyngeal samples of COVID-19 patients were identified by proteomic analysis (Akgun et al., 2020). Our observations of increased expression of alpha defensins and calprotectin in severe patients on the 1^st^ day of hospitalization are in line with previous proteomic profiling study (Meizlish et al., 2021). Of note, fatal outcome was associated with a further rise in the expression of DEFA1 and calprotectin levels in the patients with severe disease (**Figure 3E**) and 91% required mechanical ventilation. These results confirm the findings of Shi et al. (2020). Taken together, it seems that neutrophil activation signature can predict mortality in COVID-19 patients. However, there was a distinct possibility of the role of secondary infections in modulating the expression of three neutrophil secretory proteins in severe COVID-19 patients. Importantly, similar to the reports of higher proportion of secondary infection in patients from China (Zhou et al., 2020), Italy (De Santis et al., 2021), France (Roger et al., 2021) and UK (Hughes et al., 2020), 61% of severe patients from our study suffered from secondary infection. The observations of raised levels of these proteins much before the appearance of secondary infection (**Figures 3B–D**) provide important evidence for the association of higher expression levels of DEFA1/Calprotectin/MPO levels with COVID-19 severity. Moreover, the significant rise in DEFA1 levels in severe patients in comparison of patients with mild disease was independent of any confounding factors associated with disease severity (p=0.02). In our study, 53% of patients undergoing early intubation (0-3 days after hospitalization) developed secondary infection. This is in line with findings that mechanically ventilated COVID-19 patients are at high risk of developing secondary infection (Risa et al., 2021).

Multiple models have predicted neutrophils, lymphocytes, C-reactive protein (a biomarker for inflammation), and D-dimer (a measure of blood clotting) as markers for disease severity in SARS-CoV-2 infection (Zhou et al., 2020). Our findings of increased neutrophil-lymphocyte ratio along with raised D-dimer and CRP levels in the patients with severe disease confirm earlier observations (Chen et al., 2020; Liu et al., 2020; Yao et al., 2020; Zhou et al., 2020). A positive correlation was obtained between increased neutrophil-lymphocyte ratio with increased levels of DEFA1 in severe patients. A weak positive correlation between CRP and calprotectin levels was also observed in our study, probably due to small sample size. It is pertinent to note here that calprotectin was found to be superior than CRP in identifying patients requiring mechanical ventilation at any point during their hospitalization (Shi et al., 2020). Circulatory calprotectin was identified as an independent variable influencing disease severity (Silvin et al., 2020). Recognizing potential role of alpha defensins and calprotectin in augmenting inflammatory response, a clinical trial using an adjunctive therapy, cytosorb, is underway. This formulation acts by absorbing a broad spectrum of cytokines, DAMPs and PAMPs to reduce their circulatory levels and ameliorate immunopathology (Tay et al., 2020).

In summary, our data reveals that neutrophil activation as assessed by levels of circulatory alpha defensins and calprotectin varies with disease severity. DEFA1 seems to be a potential biomarker of severity, needing further evaluation. Association of raised DEFA1 and calprotectin with poor clinical outcome raises possibility of crucial roles of these molecules in the COVID-19 pathogenesis. Given the urgent need for effective treatment of COVID-19 patients, efficacy of anti-neutrophil therapies to combat neutrophil activation needs to be undertaken on priority.

## Data Availability Statement

The original contributions presented in the study are included in the article/supplementary material. Further inquiries can be directed to the corresponding author.

## Ethics Statement

The studies involving human participants were reviewed and approved by Institutional Ethics Committee of Bharati Hospital & Research Centre at Bharati Vidyapeeth Deemed University (IEC/2020/25). Informed written consent was obtained from each subject before participating in this study. This study was conducted in accordance to the ethical standards of the Helsinki Declaration of 1975, as revised in 2013. Written informed consent to participate in this study was provided by the participants’ legal guardian/next of kin.

## Author Contributions

Conceptualization, study design and supervision: VA. Methodology: SS and SC. Patient’s recruitment and clinical management: PJ, PD, and SP. Data analysis and interpretation: SS and SP. Writing of the original manuscript: SS and SC. Review and editing of the manuscript: VA and AM. All authors contributed to the manuscript and approved the submitted version.

## Funding

This work was partially supported by Indian Council for Medical Research, ICMR. The funders had no role in study design, data collection and analysis, decision to publish or preparation of the manuscript.

## Conflict of Interest

The authors declare that the research was conducted in the absence of any commercial or financial relationships that could be construed as a potential conflict of interest.

## Publisher’s Note

All claims expressed in this article are solely those of the authors and do not necessarily represent those of their affiliated organizations, or those of the publisher, the editors and the reviewers. Any product that may be evaluated in this article, or claim that may be made by its manufacturer, is not guaranteed or endorsed by the publisher.
